# Endovascular treatment of central vein occlusion in patients with functioning arteriovenous fistulas

**DOI:** 10.1590/1677-5449.210130

**Published:** 2022-03-11

**Authors:** Alex Aparecido Cantador, Lucas Lembrança Pinheiro, Ana Terezinha Guillaumon

**Affiliations:** 1 Universidade Estadual de Campinas – UNICAMP, Hospital de Clínicas – HC, Campinas, SP, Brasil.; 2 Universidade Estadual de Campinas – UNICAMP, Faculdade de Ciências Médicas – FCM, Campinas, SP, Brasil.

**Keywords:** stenosis, central vein, angioplasty, stent

## Abstract

**Background:**

The increased survival of dialysis patients and the inability to obtain sufficient organs to meet demand for transplantation, compounded by poor access to health services, have caused the transplant waiting lists to grow, extending the time spent using central venous accesses for hemodialysis. The most common etiology of central vein stenosis is prolonged central venous access, due to intimal injuries caused by the presence of the catheter.

**Objectives:**

To assess the results of angioplasty to treat central vein occlusion in patients with functioning peripheral arteriovenous fistulas.

**Methods:**

Retrospective cohort study with review of medical records from 47 patients with stenotic or occlusive lesions. Patients were assessed at 30 days, 6 months, and 1 year after recanalization or correction of stenosis with transluminal percutaneous angioplasty (TPA) or TPA/stenting.

**Results:**

Stenotic lesions were detected in 25 patients (53%) and occlusions were found in 22 (47%) patients. TPA with stenting was used in 64% of patients and balloon angioplasty in isolation was used in 36%. Analysis of clinical results showed a high rate of early clinical improvement (30 days), seen in 82% of patients (confidence interval [CI] 71-93%). After 1 year of follow-up, the primary patency rate was 57% and the assisted primary patency rate was 72% (CI 57-84%).

**Conclusions:**

Endovascular treatment of central vein stenosis or occlusions suggests clinical improvement of symptoms and adequate rates of patency at 1 year, notwithstanding the limited sample size.

## INTRODUCTION

The aging global population, the increased survival of patients with renal failure, and the shortage of organ donors have contributed to patients spending longer periods on hemodialysis. Despite the technical recommendations, it is known that poor planning and restricted access to health services (leading to delayed referral) results in the majority of patients being seen by a nephrologist at late stages. As a consequence, the great majority of patients begin renal replacement therapy with access obtained via a central venous catheter, which should only be used for short periods and as a temporary measure, but actually remains their only route for hemodialysis access for long periods. This scenario is common both in developing countries and also in those with high development indices.^1^

This situation has irreparable consequences for patients since, in addition to increasing the rate of complications, it is also the principal culprit of emergence of central vein occlusion.^2^

In this population, central vein stenosis or occlusion reaches alarming levels, with incidence rates close to 50% during renal replacement therapy reported in some series.^3^

There are several theories with regard to the origin of this condition, ranging from displacement of the rib cage during respiratory movements to intimal microlesions caused during insertion of the central catheter. Despite these disagreements, it has been proven that catheter location is the primary agent related to development of stenosis and that catheters in the subclavian vein will progress to central vein stenosis in 50% of cases.^4^

When a patient receives hemodialysis via an arteriovenous fistula (AVF) in an upper limb and has stenosis or occlusion of the ipsilateral central vein, the limb enters a state of venous hypertension, with symptoms of pain, impaired mobility, and highly significant signs of edema including clubbing, sometimes accompanied by altered perfusion of the limb and presence of ulcers associated with venous hypertension.

It should be remembered that presence of central vein stenosis in dialysis patients with functioning AVFs interferes with full hemodialysis and increases rates of admission, complications, and death. However, while the option of ligating the AVF may resolve the symptoms in the affected limb, it is not always possible to construct a new AVF (since the contralateral central veins and even those in the lower limbs may be compromised). In view of this, endovascular treatment of stenosis or occlusion of the central vein may be an option to improve the symptoms of venous hypertension and maintaining the AVF functional, particularly in patients with multiple failed accesses, while they wait for the chance of transplantation.

## OBJECTIVE

The objective of this study is to evaluate clinical improvement and patency outcomes in patients with functional peripheral AVFs who underwent angioplasty to treat central vein occlusion.

## METHODS

A retrospective cohort study was conducted with data from the medical records of 47 patients with an active AVF in an upper limb and clinical status of venous hypertension who had undergone endovascular treatment for central vein occlusion. Endovascular treatment is preferred in these cases at the service in question and treatment by ligature of the AVF is only performed in cases in which endovascular treatment fails. This study was approved by the institution’s Research Ethics Committee, CAAE number: 33327520.6.00005404, decision number 4.127.198.

The study included all dialysis patients with an active AVF in an upper limb and with clinical status of venous hypertension in the limb who had undergone endovascular treatment with angioplasty of a central vein ipsilateral to the AVF (subclavian vein or brachiocephalic trunk), with or without stenting, from January 2010 to January 2015.

**Figure 1:**
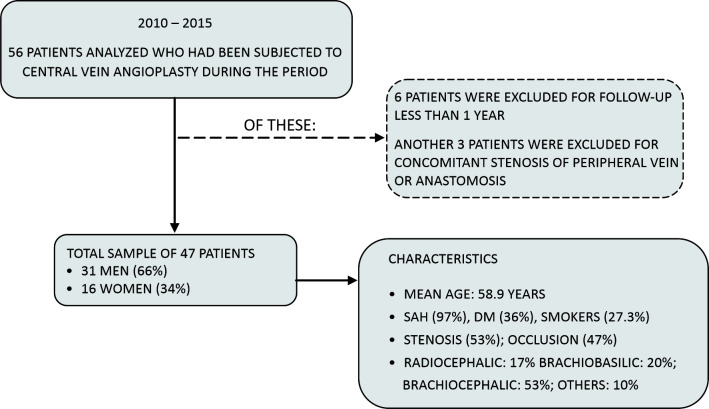
Flow diagram showing the total number of patients and the number of patients excluded together with their characteristics. SAH= systemic arterial hypertension, DM = diabetes mellitus.

Initially, 53 patients were assessed, but six were excluded from the study. The exclusion criteria were absence of follow-up for a minimum of 1 year after endovascular treatment or stenosis in the AVF anastomosis or stenosis of peripheral veins (Figure 1).

Demographic variables were analyzed and the following outcomes were defined: technical success (defined as recanalization of occlusions and absence of residual stenosis exceeding 30%), patency when followed-up with vascular echography with Doppler (at 30 days, 6 months, and 1 year), clinical improvement (defined as improvement of signs and symptoms of venous hypertension in the limb with the AVF), and reintervention rate.

The procedures were performed via venous access to the AVF limb, with insertion of a 6F introducer and peripheral and central phlebography, confirming stenosis or occlusion of the subclavian vein or the brachiocephalic vein ipsilateral to the AVF. Anticoagulation was administered with 5,000 UI of unfractionated heparin and a 0.035” hydrophilic guidewire was used to perform maneuvers to cross the lesion. Next, a high pressure balloon was used to perform angioplasty at the site of stenosis or occlusion. Control phlebography was performed after removal of the balloon and again after 15 minutes. In cases with residual stenosis exceeding 30%, angioplasty was performed again, using a self-expanding stent. Patients were discharged on the day after the procedure with prescriptions for dual antiaggregation with acetylsalicylic acid (ASA) and clopidogrel to be taken for at least 30 days.

The 47-patient sample is limited since, considering a 5% error, primary assisted patency of 72%, 82% clinical benefit, and a population of 800 patients (approximately the prevalence of central vein occlusion in the Campinas metro area), a sample size of 224 patients would be adequate to analyze assisted primary patency and 165 patients would be needed to analyze the benefit of clinical improvement.

## RESULTS

The total number of cases assessed from the period specified above was 47 patients, 31 (66%) men and 16 (34%) women, with a mean age of 58.97 years. The mean time using an AVF prior to endovascular treatment was 39 months. The mean time to endovascular treatment from onset of clinical status of venous hypertension in the limb was 10.4 months.

In this sample, 46 (97%) patients were hypertensive, 17 (36%) were diabetic, and 13 (27.3%) were smokers. There were no losses to follow-up during the study period.

Assessing the characteristics of the lesions, 25 patients (53%) had stenosis, while 22 patients (47%) had occlusions. The site of treatment was innominate veins in 57% of cases and subclavian veins in 43%. No cases of superior vena cava occlusion were treated. The site treated had no influence on improvement of symptoms or on patency.

**Figure 2:**
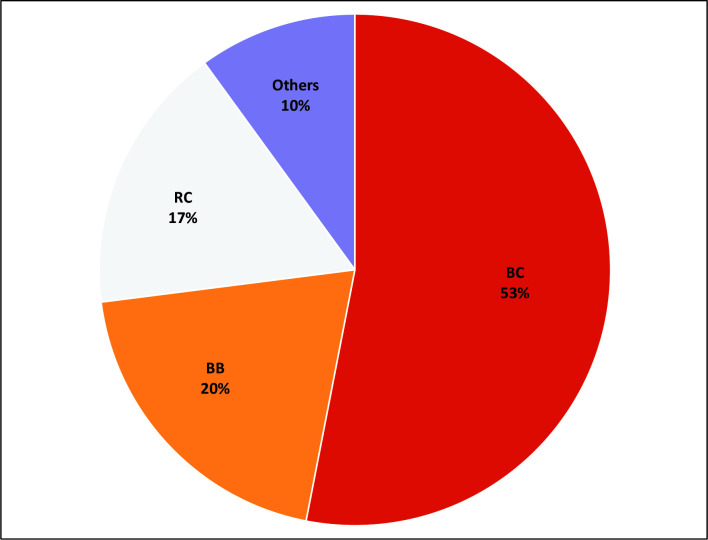
Percentages of types of AVF. RC = radiocephalic, BC = brachiocephalic, BB = brachiobasilic.

The most common type of AVF was from brachial artery to cephalic vein, in 53% of cases (Figure 2).

**Figure 3:**
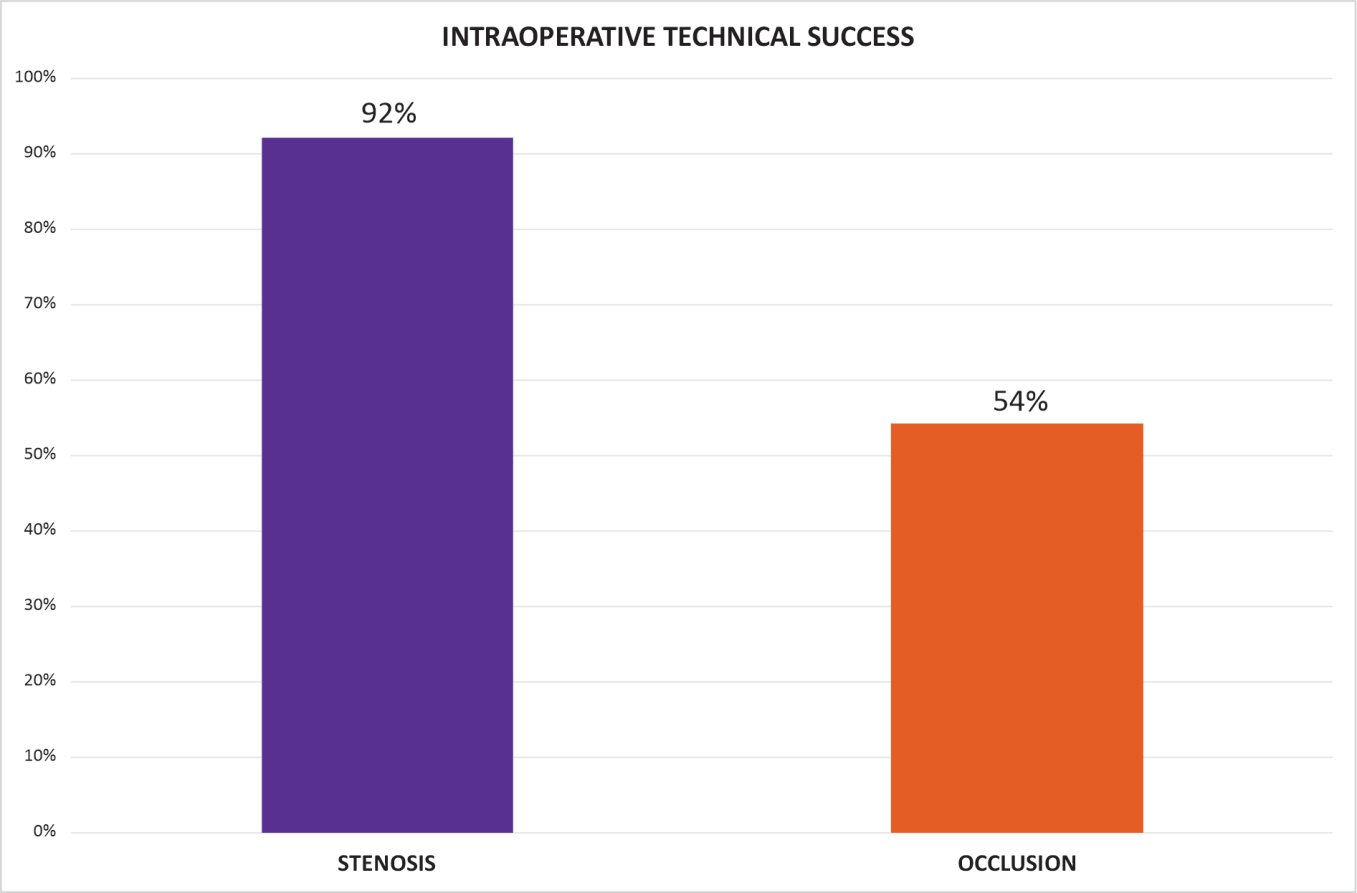
Intraoperative technical success of treatment for stenotic and occlusive lesions.

Evaluating the outcome, we observed a high technical success rate for the group with stenotic lesions (92%) and those with occlusive lesions (54%) (Figure 3).

Analyzing the types of treatment used, we observed that transluminal percutaneous angioplasty (TPA) with stenting was used in the majority of patients studied (64%) while balloon angioplasty alone was used in 36%. We observed clinical improvement of signs and symptoms in the immediate postoperative period (iPO) in 78% of cases. Of these cases that exhibited improvement in signs and symptoms of venous hypertension during the iPO, stenting was used in 73% and angioplasty alone in 27%.

Analysis of the clinical results considered improvement of signs and symptoms of edema and pain in the limb and also resolution of ulcers associated with venous hypertension in some cases, finding a high rate of early clinical improvement (30 days), in 82% of patients (confidence interval [CI] 71-93%), which remained adequate at 1 year.

**Figure 4:**
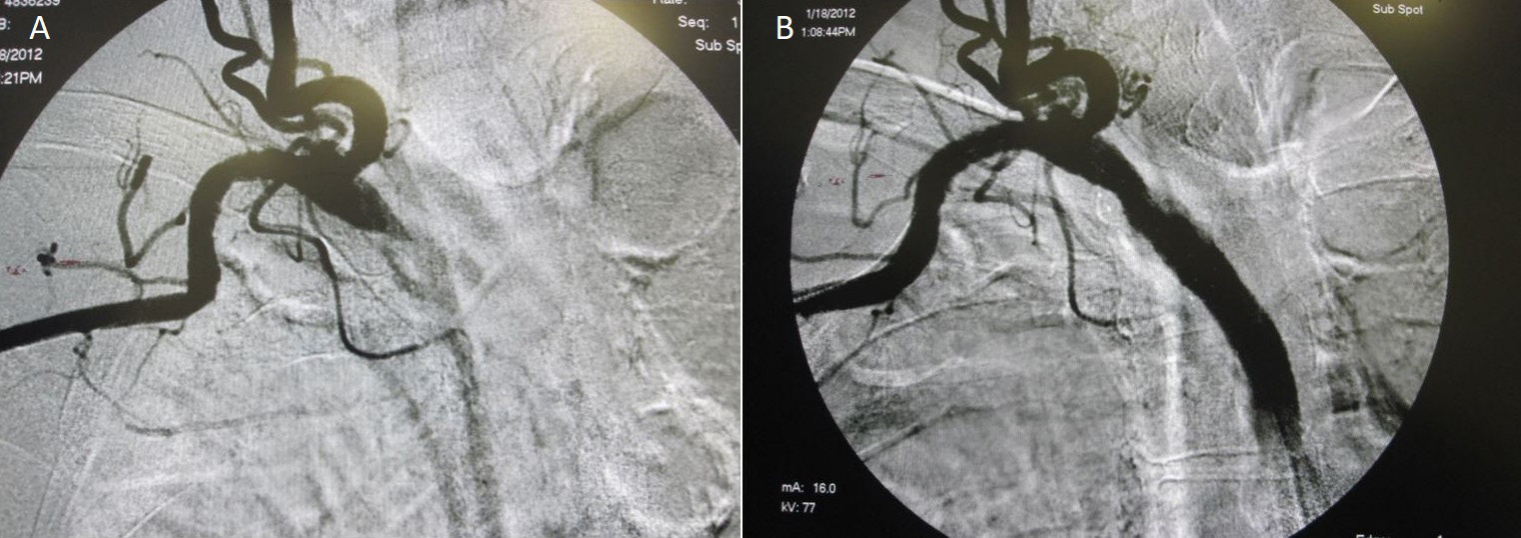
**(A)** Central vein occlusion. **(B)** Correction of central vein occlusion with endovascular technique.

Figure 4 shows an example of a case of central vein occlusion treated with success.

**Figure 5:**
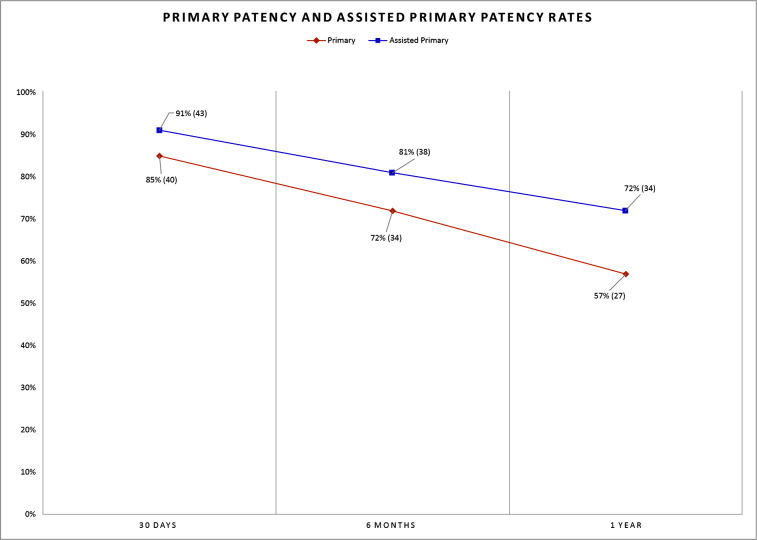
Primary patency and assisted primary patency rates according to a Kaplan-Meier plot, showing the percentage of patients and the absolute number of patients at risk of the event in parentheses.

Finally, Kaplan-Meier plots were also analyzed (Figure 5). The mean follow-up time was 16.9 months, and 11 patients (23%) needed reinterventions because of re-stenosis. At 6 months postoperative, the primary patency rate was 72% and assisted primary patency was 81%. At 1 year follow-up, the primary patency rate was 57% and the assisted primary patency rate was 72% (CI 57-84%). Postoperative follow-up was conducted with vascular echography with Doppler at 30 days, 6 months, and 1 year after treatment.

## DISCUSSION

Although central vein stenosis has a high prevalence in specific populations (dialysis patients), there are still several controversial points in relation to management of this disease. Studies of the subject use divergent definitions of stenosis and therapeutic success and there is also a lack of agreement on the methods used for imaging and follow-up of patients who undergo endovascular treatment. Nevertheless, there is consensus that endovascular treatment should always be preferred, whether with balloon angioplasty or with angioplasty and stenting. With technological improvements and new types of materials, guidewires, catheters, and stents, affording easier access and navigability in this segment, it is consensus to prefer the minimally invasive, endovascular approach. The National Kidney Foundation (NKF) recommends endovascular treatment as the first choice option for management of central vein occlusion.^4^

The characteristics of the high capacitance venous system are different from those of the arterial bed frequently treated by vascular surgeons. Stenosis of veins must be significantly greater to generate hemodynamic repercussions since drainage via collateral routes is more common. Nevertheless, these collateral veins cannot normally maintain the AVF functional and patent for long periods of time, to the extent needed to enable an adequate hemodialysis session to be completed.

Additionally, it has been proven that presence of a stent can provoke an inflammatory reaction, with cell proliferation, increasing the degree of stenosis even further in just a few months. These findings bear out the initial assertion that treatment should always be guided by symptoms or by whether the condition is compromising the dialysis process.

The definitions found in academic circles, and also in previous studies, demonstrate that primary patency and primary assisted patency rates vary widely between series and can range from 23% to 64%.^5^^,^ 6 It is therefore difficult to make a precise comparative analysis, since there is no standardization of concepts, definitions, or methodological processes between studies.

Patients treated at the reference service were investigated with a protocol employing a noninvasive pre-procedure imaging method (color Doppler ultrasonography) and treatment was only indicated if there were clinical symptoms, as mentioned above. During the endovascular operation, we started with phlebography in order to better guide correction of the stenosis and recanalizations. Initially, treatment was performed with balloon angioplasty alone and only in cases with residual stenosis exceeding 30% on control phlebographs (acquired after removal of the balloon and 15 minutes later) were self-expanding stents implanted, followed by balloon angioplasty again. At the end of the procedure, we conducted a final control angiographic examination. During the postoperative assessment of results, we again employed noninvasive imaging exams (color Doppler ultrasonography) at 1, 6, and 12 months.

Recommendations exist supporting stenting re-stenotic lesions or those in which there is residual stenosis after the first procedure.^7^ This recommendation takes into consideration the characteristics of venous lesions, which exhibit more elastic behavior, with early retraction, unlike arterial lesions, which have a more vigorous fibroelastic matrix.^8^

In a Brazilian study conducted at the Universidade Estadual de Londrina with a sample of 25 patients by Silvestre et al.,^9^ the result observed was 80% patency with reinterventions after 6 months. In an international study conducted in Belgium with a sample of 97 patients by Hongsakul et al.,^10^ the result observed was 77.3% patency with reinterventions after 6 months. Both the studies mentioned exhibited results similar to those observed in our series. Comparison of the data of this study with other series in the literature revealed that they are concordant.^6^^,^ 11 ^-^ 16

In a systematic review conducted by Xiyang Chen et al. in Washington in December 2020 analyzing twelve randomized controlled studies and four cohort studies,^17^ the authors observed significant increases in primary patency for patients treated with paclitaxel-coated balloons compared to those treated with conventional balloons. This benefit was not only observed in cases of stenosis in AVFs, but also in cases of stenosis/occlusions of central veins. The analysis of central veins was conducted on a subset of two studies.

A recent systematic review published in the Journal of Vascular Surgery: Venous and Lymphatic Disorders in September 2021 by Andrawos et al.^18^ analyzed nine case series and eight cohort studies, observing 60% overall primary patency at 1 year. Cases treated with stents exhibited superior primary patency, considering a follow-up period of up to 2 years and, in the majority of cases treated with stents (301 out of 345 patients), stenting was preceded by balloon angioplasty, with stents used in cases with residual stenosis or recoil. One significant limitation of the review identified by its authors was the absence of high quality evidence (randomized and controlled studies) available in the literature. They also suggested that adjuvant techniques such as use of intravascular ultrasound (IVUS) and drug-coated balloons and also better use of antiaggregation and anticoagulation could improve treatment patency rates.

According to the most recent Kidney Disease Outcomes Quality Initiative (KDOQI) guidelines,^19^ asymptomatic cases of central vein stenosis or occlusion should not be treated because angioplasty in asymptomatic patients is associated with more rapid progression to symptomatic stenosis. Primary use of stents is not recommended in cases of central vein stenosis/occlusion and balloon angioplasty is indicated as initial intervention. High pressure balloons and covered stents are indicated for stenosis of peripheral veins, but there is no evidence of superiority of either high pressure balloons or covered stents for stenosis/occlusions of central veins. The HeRO device (Hemoaccess Reliable Outflow, Hemosphere, Inc, Kennesaw, GA) is a hybrid alternative option (endovascular and conventional surgery) that is normally used for new accesses, but can also be used in combination with an existing AVF associated with central vein occlusion.

Although the limited sample of this study conducted at our service precludes extrapolation as recommendations for other centers, and considering that the KDOQI guidelines have an adequate level of evidence for current recommendations, the present study reports satisfactory results that are in line with those of other centers, providing a reflection of the advantages of endovascular treatment in cases of central vein occlusion. With developments in the field of endovascular devices (for example, the HeRO device, IVUS, and paclitaxel-coated balloons), in the future it will be possible to assess the results of new technologies, comparing them with conventional balloon angioplasty.

## CONCLUSIONS

Endovascular treatment of central vein stenosis or occlusions suggests clinical improvement of symptoms and adequate patency rates after 1 year, notwithstanding the limited sample size. Treatment is of low invasivity and restores AVF functionality, reducing edema and the complications inherent to upper limb venous hypertension. Preservation of the AVF is extremely important for patient survival.
