# Supplementary material for: Endovascular treatment of central vein occlusion in patients with functioning arteriovenous fistulas
Source: J Vasc Bras. 2022 Mar 11;21:e20210130. doi: 10.1590/1677-5449.210130 (PMC8958434; doi:10.1590/1677-5449.210130)
Supplement: Translation

# Translation: Tratamento endovascular de doença oclusiva venosa central com fístula arteriovenosa funcionante

**Authors:** Alex Aparecido Cantador, Lucas Lembrança Pinheiro, Ana Terezinha Guillaumon

**Affiliations:** 1 Universidade Estadual de Campinas – UNICAMP, Hospital de Clínicas – HC, Campinas, SP, Brasil.; 2 Universidade Estadual de Campinas – UNICAMP, Faculdade de Ciências Médicas – FCM, Campinas, SP, Brasil.

**Keywords:** estenose, veia central, angioplastia, stent

## Abstract

**Contexto:**

A maior sobrevida dos doentes dialíticos somada à incapacidade de obtenção de órgãos suficientes para atender a demanda, bem como à dificuldade de acesso aos serviços de saúde, levou ao aumento da fila para transplante e ao prolongamento do tempo de utilização do acesso venoso central para hemodiálise. A etiologia mais comum de estenose de veia central é o acesso venoso central prolongado, pelas lesões intimais decorrentes da presença do cateter.

**Objetivos:**

Avaliar resultados de angioplastia para tratamento de doença oclusiva venosa central com fístula arteriovenosa periférica funcionante.

**Métodos:**

Estudo tipo coorte retrospectivo com revisão de prontuários de 47 doentes com lesões estenóticas ou oclusivas. A avaliação dos doentes foi realizada em 30 dias, 6 meses e 1 ano após a recanalização ou correção da estenose com ATP ou ATP/aplicação de stent.

**Resultados:**

Lesões estenóticas foram encontradas em 25 doentes (53%), e oclusões, em 22 (47%) doentes. A angioplastia percutânea transluminal (ATP) com stent foi utilizada em 64% dos doentes, e angioplastia isolada com balão, em 36% deles. A análise de resultados clínicos mostrou elevada taxa de melhora clínica precoce (30 dias) em 82% dos doentes (intervalo de confiança [IC] 71-93%). Após 1 ano de seguimento, a taxa de perviedade primária foi de 57%, e a taxa de perviedade primária assistida foi de 72% (IC 57-84%).

**Conclusão:**

O tratamento endovascular das estenoses ou oclusões de veia central sugere melhora clínica dos sintomas e taxas adequadas de perviedade no período de 1 ano, apesar da limitação no tamanho amostral.

## INTRODUÇÃO

O envelhecimento global da população, o aumento da sobrevida de doentes com insuficiência renal e a insuficiente doação de órgãos levaram ao aumento do tempo em que os doentes são submetidos à hemodiálise. Apesar das recomendações técnicas, sabe-se que, por falta de planejamento e acesso aos serviços de saúde, com referência inadequada, a maior parte dos doentes chega tardiamente ao nefrologista. Dessa forma, a grande maioria inicia sua terapia renal substitutiva através de um cateter venoso central, que deveria ser utilizado por um período curto e temporário, porém permanece por muito tempo como único acesso para hemodiálise. Esse fato é comum tanto em países em desenvolvimento como naqueles com altos níveis de desenvolvimento^1^.

Essa realidade traz consequências irreparáveis para esses doentes, na medida em que, além de aumentar a taxa de complicações relacionadas ao acesso, é a principal responsável pela gênese da doença oclusiva venosa central^2^.

Nessa população, a estenose ou oclusão de veia central chega a níveis alarmantes, sendo reportada, em algumas séries, incidência próxima de 50% durante a terapia renal substitutiva^3^.

Existem diversas teorias quanto à origem dessa condição, desde mobilização da caixa torácica em função dos movimentos respiratórios, como microlesões intimais causadas durante a inserção do cateter central. Apesar das controvérsias, é comprovado que a localização do cateter é o principal agente relacionado com o desenvolvimento da estenose e que o cateter em veia subclávia evolui para estenose da veia central em 50% dos casos^4^.

Quando o doente realiza hemodiálise por fístula arteriovenosa (FAV) no membro superior e apresenta estenose ou oclusão de veia central ipsilateral, ocorre uma situação de hipertensão venosa no membro, com sintomas de dor e alterações de mobilidade e sinais de edema muito significativo com empastamento do membro, além de, eventualmente, alterações da perfusão do membro e presença de úlceras associadas à hipertensão venosa.

Devemos considerar que a presença de estenose de veia central em doentes dialíticos com FAV funcionante dificulta a realização da hemodiálise plena e aumenta as taxas de internação, complicações e óbito. Por outro lado, a alternativa de realizar a ligadura da FAV pode resolver os sintomas no membro acometido, porém nem sempre é factível a confecção de uma nova FAV (pode haver comprometimento das veias centrais contralaterais e até dos membros inferiores). Dessa forma, o tratamento endovascular da estenose ou oclusão da veia central pode melhorar os sintomas da hipertensão venosa e manter a FAV funcionante, principalmente para os doentes com falência múltipla de acessos, enquanto aguardam possibilidade de transplante.

## OBJETIVO

Este estudo teve como objetivo avaliar resultados de melhora clínica e perviedade em pacientes submetidos à angioplastia para tratamento de doença oclusiva venosa central com FAV periférica funcionante.

## MÉTODOS

Estudo tipo coorte retrospectiva com levantamento de prontuários de 47 doentes com FAV ativa em membro superior e quadro clínico de hipertensão venosa, submetidos a tratamento endovascular de doença oclusiva venosa central. O tratamento endovascular nesses casos é o preferido no serviço, sendo realizado o tratamento de ligadura da FAV somente nos casos de falha do tratamento endovascular. Este estudo foi aprovado pelo Comitê de Ética em Pesquisa da Instituição, número CAAE: 33327520.6.0000.5404, parecer número 4.127.198.

Foram incluídos todos os pacientes dialíticos e com FAV ativa em membro superior, com quadro clínico de hipertensão venosa no membro, submetidos a tratamento endovascular com angioplastia de veia central (v. subclávia ou tronco braquiocefálico) ipsilateral à FAV, com utilização de stent ou não, no período de janeiro de 2010 a janeiro de 2015.

Inicialmente, foram avaliados 53 doentes, sendo seis excluídos do estudo. Os critérios de exclusão foram ausência de seguimento pelo período mínimo de 1 ano após o tratamento endovascular ou presença de estenoses na anastomose da FAV ou estenose das veias periféricas (Figura 1).

**Figura 1:** Fluxograma mostrando o número total de pacientes e o número de pacientes excluídos, assim como suas características. HAS = hipertensão arterial sistêmica, DM = diabetes melito.

Foram analisadas as variáveis demográficas e definidos os seguintes desfechos: sucesso técnico (definido pela recanalização das oclusões e ausência de estenose residual maior que 30%), perviedade ao acompanhamento com ecografia vascular com Doppler (períodos de 30 dias, 6 meses e 1 ano), melhora clínica (definida pela melhora dos sinais e sintomas de hipertensão venosa no membro da FAV) e taxa de reintervenções.

Os procedimentos foram realizados através de acesso venoso no membro da FAV, com inserção de introdutor 6F e realização de flebografia periférica e central, confirmando a estenose ou oclusão da veia subclávia ou da veia inominada ipsilateral à FAV. Realizou-se anticoagulação com 5.000 UI de heparina não fracionada, e, utilizando fio guia hidrofílico 0,035”, foram realizadas manobras para ultrapassar a lesão. Em seguida, foi utilizado balão de alta pressão para realização da angioplastia no local acometido pela estenose ou oclusão. A flebografia de controle foi realizada após retirada do balão e após 15 minutos. Nos casos de estenose residual superior (30%), foi realizada nova angioplastia com utilização de stent autoexpansível. Os pacientes receberam alta no dia seguinte ao procedimento com orientação de uso de dupla antiagregação plaquetária com ácido acetilsalicílico (AAS) e clopidogrel durante pelo menos 30 dias.

A amostra de 47 doentes é limitada, sendo que, considerando 5% de erro para perviedade primária assistida de 72% e benefício clínico de 82% e considerando uma população de 800 doentes (aproximadamente a prevalência de doença oclusiva venosa central na região metropolitana de Campinas), a amostra adequada seria de 224 doentes para análise da perviedade primária assistida e 165 doentes para análise do benefício de melhora clínica.

## RESULTADOS

O número total de casos avaliados no período supracitado foi de 47 doentes, sendo 31 (66%) homens e 16 (34%) mulheres, com média de idade 58,97 anos. O tempo médio de utilização da FAV previamente ao tratamento endovascular foi de 39 meses. O tempo médio anterior ao tratamento endovascular de início do quadro clínico de hipertensão venosa no membro foi de 10,4 meses.

Nesta amostra, 46 (97%) eram hipertensos, 17 (36%) eram diabéticos e 13 (27,3%) eram tabagistas. Não houve perda de seguimento no período estudado.

Avaliando-se as características das lesões, 25 doentes (53%) apresentavam estenoses, enquanto 22 doentes (47%) apresentavam oclusões. Os locais de tratamento foram as veias inominadas em 57% dos casos e veias subclávias em 43%. Não foram tratados casos de oclusão da veia cava superior. Não houve influência do local tratado em relação à melhora dos sintomas ou em relação à perviedade.

O tipo de FAV mais comum foi entre a artéria braquial e a veia cefálica, em 53% dos casos (Figura 2).

**Figura 2:** Porcentagem do tipo de FAV. RC = radiocefálica, BC = braquiocefálica, BB = braquiobasílica.

Ao avaliarmos o desfecho, obtivemos alta taxa de sucesso técnico para o grupo de lesões estenóticas (92%) e de lesões oclusivas (54%) (Figura 3).

**Figura 3:** Sucesso técnico das lesões estenóticas e oclusivas no intraoperatório.

Analisando o tipo de tratamento empregado, verificamos que foi realizada angioplastia percutânea transluminal (ATP) com colocação de stent na maioria dos doentes estudados (64%) e angioplastia isolada com balão em 36% deles. Verificamos melhora clínica dos sinais e sintomas em 78% dos casos no pós-operatório imediato (POi), sendo que, dos casos que apresentaram melhora dos sinais e sintomas da hipertensão venosa no POi, foi utilizado stent em 73% e angioplastia isolada em 27%.

A análise de resultados clínicos, sendo considerada a melhora dos sinais e sintomas de edema e dor no membro, bem como a resolução de úlceras associadas à hipertensão venosa em alguns casos, mostrou elevada taxa de melhora clínica precoce (30 dias) em 82% dos doentes (intervalo de confiança [IC] 71-93%), permanecendo adequada ao longo do período de 1 ano.

Podemos observar, na Figura 4, um exemplo de caso de oclusão de veia central tratado com sucesso.

**Figura 4:** **(A)** Oclusão de veia central. **(B)** Correção da oclusão de veia central pela técnica endovascular.

Por fim, foram analisadas as curvas de Kaplan-Meier (Figura 5). O tempo médio de seguimento foi de 16,9 meses, sendo que, em 11 doentes (23%), houve a necessidade de reintervenções por re-estenoses. Considerando o tempo pós-operatório de 6 meses, obtivemos taxa de perviedade primária de 72% e de perviedade primária assistida de 81%. Após 1 ano de seguimento, a taxa de perviedade primária foi de 57%, e a taxa de perviedade primária assistida foi de 72% (IC 57-84%). O acompanhamento pós-operatório foi realizado com ecografia vascular com Doppler nos períodos de 30 dias, 6 meses e 1 ano após o tratamento.

**Figura 5:** Taxas de perviedade primária e primária assistida conforme curva de Kaplan-Meier, mostrando o percentual de pacientes e o número absoluto de pacientes em risco de evento entre parênteses.

## DISCUSSÃO

Apesar de a estenose de veia central apresentar elevada prevalência em populações específicas (doentes dialíticos), ainda existem diversos pontos controversos no manejo dessa doença. Os trabalhos que abordam o tema apresentam definições diversas quanto à definição de estenose e de sucesso terapêutico, bem como sobre o uso de método discordante no que diz respeito à avaliação por imagem e seguimento dos doentes submetidos ao tratamento endovascular. Contudo, é consenso que o tratamento endovascular deve ser sempre o preferido, seja por angioplastia com balão ou angioplastia e stent. Com a melhora tecnológica dos novos tipos de materiais, fios guias, cateteres e stents, com mais fácil acesso e navegabilidade nesse segmento, é consenso preferir a abordagem minimamente invasiva, endovascular. A *National Kidney Foundation* (NKF) recomenda o tratamento endovascular como primordial na abordagem da doença oclusiva venosa central^4^.

A característica do sistema venoso de alta capacitância é diferente do leito arterial frequentemente abordado por cirurgiões vasculares. A estenose de veias precisa ser significativamente maior para gerar repercussão hemodinâmica, pois o desague por vias colaterais é mais frequente. Apesar disso, essas veias colaterais normalmente não conseguem manter a FAV funcionante e pérvia por longo período de tempo, de forma a permitir uma sessão de hemodiálise adequada.

Além disso, já foi comprovado que a presença do stent pode provocar uma inflamação reacional, com proliferação celular, aumentando ainda mais o grau de estenose em poucos meses. Tais achados ratificam a assertiva inicial de que o tratamento deve ser sempre guiado pelos sintomas ou se estiver comprometendo o processo de diálise.

As múltiplas definições presentes no meio acadêmico, bem como nos estudos prévios, demonstram que as taxas de perviedade primária e primária assistida variam bastante entre as séries, podendo variar de 23% a 64%^5^^,^ 6. Dessa forma, há dificuldade em uma análise comparativa precisa, pois não existe uma padronização de conceitos, definições e processos metodológicos entre os estudos.

Os doentes tratados no serviço seguiram protocolo de investigação através de método de imagem não invasivo (eco-Doppler colorido) pré-procedimento, e somente na presença de sintomas clínicos, conforme mencionado previamente, foi definida a indicação do tratamento. No ato operatório pela técnica endovascular, iniciamos o mesmo com uma flebografia para condução melhor na correção das estenoses e das recanalizações. Foi realizado inicialmente o tratamento com angioplastia com balão, e, nos casos de estenose residual maior que 30% em flebografias de controles (realizadas após retirada do balão e após 15 minutos), foram implantados stents autoexpansíveis, seguidos de nova angioplastia com balão após. Ao final do procedimento, realizamos exame angiográfico final de controle. No período pós-operatório de avaliação do resultado, utilizamos novamente exame de imagem não invasivo (eco-Doppler colorido) após 1, 6 e 12 meses.

Existe recomendação no sentido do implante de stent para lesões re-estenóticas e na presença de estenose residual quando da realização do primeiro procedimento^7^. Tal recomendação leva em consideração as características das lesões venosas que apresentam comportamento mais elástico com retração precoce, diferentemente das arteriais que apresentam matriz fibroelástica mais vigorosa^8^.

No trabalho nacional realizado na Universidade Estadual de Londrina, por Silvestre et al.^9^, com amostra de 25 doentes, o resultado obtido foi de 80% de perviedade com reintervenções após 6 meses. No trabalho internacional realizado na Bélgica, por Hongsakul et al.^10^, com amostra de 97 doentes, o resultado obtido foi de 77,3% de perviedade com reintervenções após 6 meses. Ambos os trabalhos mencionados apresentaram resultados semelhantes aos observados em nossa série. Ao compararmos os dados deste estudo com outras séries da literatura, verificamos que são concordantes^6^^,^ 11 ^-^ 16.

Em uma revisão sistemática realizada em Washington, em dezembro de 2020, por Xiyang Chen et al.^17^, englobando doze estudos randomizados e controlados e quatro estudos tipo coorte, os autores observaram aumento significativo da perviedade primária para os doentes tratados com balão revestido com paclitaxel em relação aos balões convencionais. Esse benefício foi observado não apenas nos casos de estenoses em FAVs, mas também nos casos de estenoses/oclusões em veias centrais. Essa análise das veias centrais foi realizada considerando um subgrupo de dois estudos.

Em uma revisão sistemática recente, publicada em setembro de 2021 no Journal of Vascular Surgery: Venous and Lymphatic Disorders por Andrawos et al.^18^, foram analisadas nove séries de casos e oito estudos tipo coorte, com resultados de patência primária geral de 60% em 1 ano. Os casos tratados com stent apresentaram superioridade na patência primária, considerando um período de até 2 anos de seguimento, sendo que a maioria dos casos tratados com stent (301 de 345 doentes) foi precedida de angioplastia com balão, sendo utilizado stent nos casos de estenose residual ou recoil. Como limitação significativa da revisão, os autores apontaram a ausência de evidências de alta qualidade (estudos randomizados e controlados) disponíveis na literatura. Também sugeriram que técnicas adjuvantes como a utilização de ultrassom intravascular (IVUS) e balões recobertos com drogas, assim como melhor utilização de terapia antiagregante plaquetária e anticoagulação poderiam melhorar as taxas de patência no tratamento.

De acordo com a diretriz mais recente da *Kidney Disease Outcomes Quality Initiative* (KDOQI)^19^, nos casos de estenose ou oclusão de veia central assintomática, não deve ser realizado o tratamento, pois a angioplastia no doente assintomático está associada a progressão mais rápida para estenose sintomática. A utilização primária de stents nos casos de estenose/oclusão venosa central não é recomendada, sendo indicada a angioplastia com balão inicialmente. Os balões de alta pressão e os stents revestidos são indicados nas estenoses de veias periféricas, porém, para estenoses/oclusões de veias centrais, não existe evidência de superioridade tanto dos balões de alta pressão como dos stents revestidos. O dispositivo HeRO (Hemoaccess Reliable Outflow, Hemosphere, Inc, Kennesaw, GA) consiste em alternativa híbrida (endovascular e cirurgia convencional), sendo normalmente utilizado para novos acessos, e também pode ser utilizado combinado com uma FAV existente associada a doença oclusiva venosa central.

Apesar da amostra limitada no estudo desenvolvido em nosso serviço, não permitindo extrapolação para recomendação de conduta para outros centros, e considerando a diretriz KDOQI, que apresenta nível adequado de evidência nas recomendações atuais, o presente trabalho demonstra resultados satisfatórios e condizentes com outros centros, permitindo uma reflexão sobre as vantagens do tratamento endovascular nos casos de doença oclusiva venosa central. Com o avanço dos dispositivos endovasculares (por exemplo, o dispositivo HeRO, o IVUS e os balões revestidos com paclitaxel), será possível avaliar futuramente os resultados das novas tecnologias, realizando comparações com a angioplastia com balão convencional.

## CONCLUSÃO

O tratamento endovascular das estenoses ou oclusões de veia central sugere melhora clínica dos sintomas e taxas adequadas de perviedade no período de 1 ano, apesar de limitação no tamanho amostral. O tratamento é pouco invasivo, restaurando a funcionalidade das FAVs e reduzindo o edema e as complicações inerentes à hipertensão venosa dos membros superiores. A preservação da FAV é de extrema importância para a sobrevida do doente.
